# Psychological interventions for improvement of symptoms of post-stroke depression – study protocol of the depression-intervention study for optimization of reconvalesence after stroke (DISCOVER)

**DOI:** 10.1186/s42466-024-00347-y

**Published:** 2024-12-11

**Authors:** Lino Braadt, Simone Fischer, Markus Naumann, Philipp Zickler, Thomas Schneider-Axmann, Laura Mühlich, Katharina Körber, Alexander Lassner, Wolfgang Strube, Astrid Röh, Alkomiet Hasan, Michael Ertl

**Affiliations:** 1 Neurology, Faculty of Medicine, University of Augsburg, Stenglinstrasse 2, 86156 Augsburg, Germany; 2 Epidemiology, Faculty of Medicine, University of Augsburg, Stenglinstrasse 2, 86156 Augsburg, Germany; 3 Department of Psychiatry, Psychotherapy and Psychosomatics, Faculty of Medicine, University of Augsburg, Geschwister-Schönert-Str. 1, 86156 Augsburg, Germany; 4 Department of Psychiatry and Psychotherapy, University Hospital, LMU Munich, Munich, Germany; 5 DZPG (German Center for Mental Health), Partner Site München/Augsburg, Augsburg, Germany

**Keywords:** Post-stroke depression, Telepsychological intervention, Rehabilitation, Stroke

## Abstract

**Introduction:**

Besides functional outcomes, mental health and health-related quality of life (HRQoL) are significant measures in chronic diseases, such as stroke. Post-stroke depression (PSD) is an important complication affecting up to one in three stroke survivors. So far, specific programs to screen, detect and treat these patients are lacking but might be a crucial component in stroke aftercare.

**Methods:**

Between March 2024 and Febuary 2025 all consecutive adult patients with stroke, admitted to the University Hospital of Augsburg, are screened for signs of depression (PHQ-9 (Patient Health Questionnaire-9) score of ≥ 10). Eight weeks later, those patients with persisting signs of depression will be randomized to receive either a behavioural activation intervention or a mindfulness-based intervention via telephone. Depressive symptoms will be assessed at baseline (V1), after 84, 180 (V2-V3) and after 365 days (V4) using the PHQ-9 score. Secondary outcomes include the functional outcome, quality of life (EuroQol-5 Dimensions; EQ-5D), overall functioning, and incidence of major cardiovascular events including recurrent transient ischemic attack, stroke, and mortality.

**Perspective:**

Our aim is to provide proof-of-concept evidence for the efficacy of telepsychological need-adapted interventions for patients with post-stroke depression. Regular screening for PSD with implementation of a feasible treatment option could reduce the long-term prevalence of PSD and improve quality of life.

**Trial registration:**

This trial was registered at German Clinical Trials Register (DRKS) on 07.03.2024, ID: DRKS00033792. German Clinical Trials Register (drks.de).

## Introduction

Globally there were 12.2 million incident and 101 million prevalent cases of stroke in 2019. Thereby, stroke caused 143 million disability-adjusted life-years (DALYs) [1]. Assessing the functional status and disability after stroke is important, but stroke severity measures, such as the National Institutes of Health Stroke Scale (NIHSS), do not capture other important aspects well, such as health-related quality of life (HRQoL) [2]. Major limitations of HRQoL after stroke are neuropsychological disorders, especially post-stroke depression (PSD). Although often being overlooked [3] PSD is already one of the most common neuropsychological complications after stroke, affecting up to 31% of stroke survivors [4] and may also have an increasing incidence and prevalence in the future. Hence, effective, and resource-efficient treatment options are needed.

PSD has a complex impact. It is associated with a poorer recovery of activities of daily living (ADL) [5]. It also affects, among other factors such as stroke severity, ethnicity and socioeconomic status, the return to work rate, which ranged from 28 to 88% among stroke patients [6]. At the same time, the prevalence of depression and anxiety is higher in patients who do not return to work [7]. Therefore, PSD is not just a factor that reduces HRQoL, but is also a health- and socio-economic issue that is associated with higher impairment of ADL, less successful rehabilitation, and lower return to work rates.

Current treatment options include pharmacotherapy, psychotherapy, electroconvulsive therapy, hyperbaric oxygen therapy and neuromodulation [3]. Pharmacotherapy is part of standard treatments, also being recommended in guidelines [8], but there is not only conflicting evidence about specific drug class superiority [8] but also contradictory evidence about its effectiveness in general [3]. Besides this rather weak evidence, it is known that antidepressants may have severe adverse outcomes, such as increased mortality in stroke survivors with depression, gastrointestinal bleeding, falls and factures [9]. Such risks are not present in psychological therapy of PSD. Nevertheless, a recent Cochrane review found, that there is very low certainty of evidence, that psychological therapy may reduce the incidence of PSD at the end of treatment. This was mainly attributed to the methodology of the respective trials [10]. The treatment methods ranged from problem-solving therapy, cognitive behavioural coping therapy [11] to a “solution-focused brief therapy” [12] and a “motivational interviewing” [13]. Participants were treated for four weeks up to 52 weeks [10]. Another study examined repeated psychosocial-behavioural interventions with drug treatment versus drug treatment only [14]. Less patients met the study criteria for depression at the end of each treatment [10, 14]. These treatments were carried out in a direct person-professional interaction, thus limiting the accessibility. Telepsychological interventions, delivered remotely, can overcome this barrier, thus being resource-efficient treatment options, eliminating the need for patients with disability after stroke or therapists to travel. Furthermore, they improve scalability in times of growing demands. Telepsychological interventions have been studied in general and video conferenced mental and behavioural health services already showed promising results in patients with depression [15].

To overcome the downsides of pharmacotherapy and of conventional psychotherapy, we examine the short- and long-term efficacy of telepsychological Behavioural Activation (BA) therapy for patients with PSD in this monocentric randomized and controlled rater-blinded study. BA represents an innovative and evidence-based advancement of classical behaviour activation. It integrates techniques from Acceptance and Commitment Therapy (especially value work) and Dialectical Behavioural Therapy (particularly validation techniques and emotion regulation strategies) in a highly structured and easily learnable manner. Stemming from the reinforcement loss model of depression, its core focus lies in planning, implementing, and rebuilding value-oriented behaviour, enabling patients to gain long-term, stable access to positive reinforcement. The control group will receive guided mindfulness-based practices [16].

Identification of patients with PSD is a crucial part of PSD management, because it is frequently overlooked even though its high prevalence. Screening for PSD helps to avoid neglecting patients who require care, but also conserves resources by not having to treat every patient with stroke preventively, as it has been suggested in previous studies.

We hypothesize that telepsychological interventions are feasible and effective treatment options for PSD, thus contributing to rehabilitation outcomes and overall improvement of HRQoL after stroke.

## Methods

### Aim of the trial

The presented interventional study is part of the digiBRAVE (digitale Bayerische (Früh-)-Diagnostik-, Prävention und Therapieprogramm Depression) program, and will be carried out at the University Hospital of Augsburg and remotely. DISCOVER is designed as a proof-of-concept study, since this is the first concept of using Behavioural Activation (BA) therapy vs. a control group to treat depressive symptoms in patients with stroke.

The Department of Neurology at the University Hospital Augsburg is a tertiary stroke center and the sole hospital providing acute stroke care for all stroke patients in the city of Augsburg and the surrounding county (around 750,000 people in total), which helps to minimize selection bias. Approximately 1,800 patients with acute stroke are treated each year at the University Hospital of Augsburg.

### Study description and study design

We compare the efficacy of telepsychological Behavioural Activation (BA) therapy with guided mindfulness-based practices for patients with PSD in this monocentric, randomized, controlled, rater-blinded trial. The allocation ratio is 1:1. Repeated follow-ups are planned up to one year after enrolment to assess short- and long-term effects.

Baseline characteristics such as age, sex, cerebrovascular risk factors, history of relevant diseases, history of preceding strokes, stroke etiology, stroke severity (NIHSS and modified Rankin scale (mRS)) at admission and discharge and acute stroke treatment will be gathered from the patients’ chart.

Following the screening visit (V0), patients with persisting depressive symptoms after eight weeks (56 days) at the baseline visit (V1) are randomized either to the intervention or the control condition and are invited to three more visits until the end of study visit (V4, day 365). The primary outcome is evaluated at visit 2 (day 84), the secondary outcomes at the end-of study visit (V4). Table 1 shows the detailed scope and the frequency of study visits.

**Table 1:**
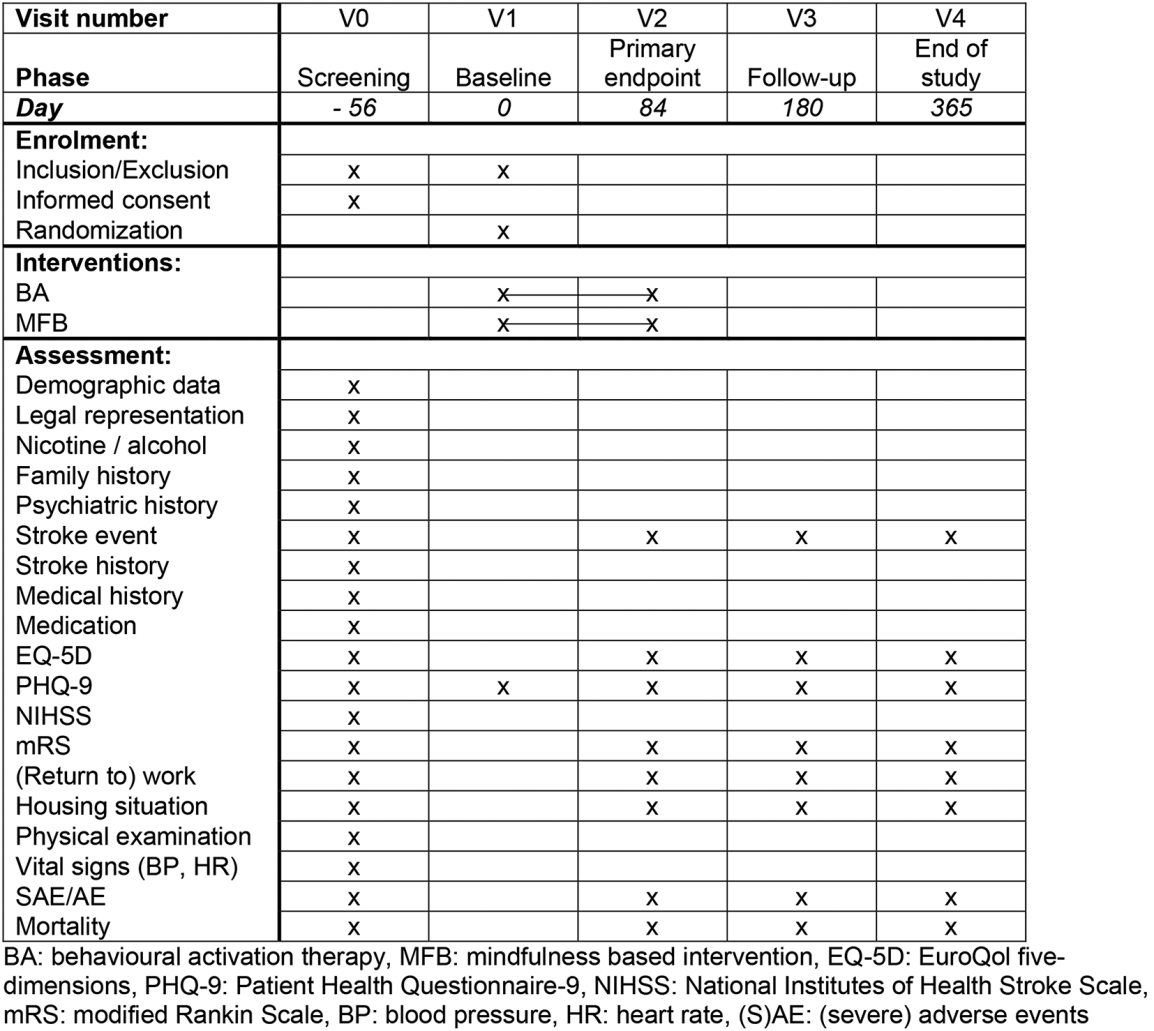
Schedule of enrolment, interventions, and assessments

We use an external randomization performed by a party not involved in the recruitment and treatment of patients. Patient selection will follow a systematic strategy and is documented according to CONSORT. The randomization list will be produced by a statistician not involved in the study.

For the primary outcome (of depressive symptoms between baseline and day 84) assuming repeated measures ANOVA as analysis method, a type I error probability of α = 0.05, a power of 1- β = 0.8, two groups at V2 (day 84), and a correlation between the measurements of *r* = 0.5, medium effects of f = 0.30 or higher can be detected for the within-subject factor time, for the between-subject factor group, and for interactions between time and group with a total necessary sample size of 68 (34 participants per group) at V2. No previous data is available to estimate the expected effect size. These calculations were performed with G*Power 3.1.9.7 [17].

The intention-to-treat (ITT) population includes all patients as randomized, and the modified intention-to-treat (mITT) population includes all patients as randomized + having received the first visit after signed informed consent (baseline visite). The safety population includes all patients as randomized. Assuming a drop-out rate of 10% during the intervention period, 76 patients are planned to be randomized. The per protocol (PP) population will include all participants without major protocol violations. All primary analyses will be performed on the ITT population. For the primary endpoint, a linear mixed model will be performed and in cases of significant ‘group x time’ interactions, post-hoc comparisons corrected for multiple testing will be performed. Continuous secondary endpoints will be analysed using linear mixed models and subsequent post-hoc tests if the requirements for this strategy are met (tested by Kolmogorov-Smirnov tests and Levene’s tests). In cases where the assumptions of normality or variance homogeneity are violated, a monotonic transformation of variables will be performed. If this first step is not successful, corresponding non-parametric tests will be used. Side-effects, adverse events (AE) and severe adverse events (SAE) will be analysed with descriptive statistics and likelihood-ratio tests. Demographic information will be shown for each group separately, and they will be compared between the groups. Dichotomous variables will be analysed with likelihood-ratio tests and continuous variables will be analysed using analyses of variance or respective non-parametric tests. Baseline characteristics are presented as mean (M) ± standard deviation (SD) for normal distributed and additionally median and 25th and 75th quartiles (Q25, Q75) for all continuous variables. Categorical variables are given as numbers (n) and percentages (%). An interim analysis is not planned. The statistician will be blinded for the groups during the phase of data-analyses. Prior to the start of the trial, a statistical analysis plan will be developed.

All analyses will be performed at a significance level of α = 0.05.

### Arms and interventions

Upon enrolment into the study, both the intervention and control groups receive leaflets with information about PSD, along with the advice to seek help, via emergency phone numbers or websites, if they have such symptoms. Patients in the interventional group receive the active intervention, patients without confirmation of a depressive episode are assigned to the intention to treat group. Intervention and control groups receive weekly phone calls over a period of 12 weeks, at the earliest time point of eight weeks after the acute stroke.

The intervention group receives a Behavioural Activation (BA) therapy. The intervention follows the BA procedure used in the Change PDD study (a psychotherapy study funded by DFG (Deutsche Förderungsgemeinschaft)). In each phone call, techniques will be taught, structured, and attempted to be integrated into the patients’ daily lives in an accessible and gradual manner. The phone calls last 25 min and are conducted by BA-trained professionals with access to regular supervision by BA-certified psychologists.

The control group is contacted at the same time as the intervention group after the index event. At 84, 180 and 365 days after inclusion standardized postal questionnaires are sent to the patients. The phone contacts match the frequency and scope of the intervention group. The control group will receive guided mindfulness-based practices conducted by trained professionals.

Irrespective of the allocation, all patients will receive a state-of-the-art treatment according to the guidelines of secondary stroke prevention. They receive counselling and education on stroke pathophysiology, risk factor management, lifestyle improvement and medication compliance by a stroke specialist. Only selected high-risk patients are seen in the outpatient clinic of the stroke center. The patient’s general practitioner manages post discharge risk-factor management. This also covers the recommended therapies for comorbidities after stroke, such as post stroke depression, fatigue etc. Additional treatments are not part of the study.

The above-mentioned interventions are unlikely to cause any somatic side effects. Mental decompensations are conceivable. To address this, we provide the patients with an emergency contact (phone number), which will be available 24/7. We hypothesize, that the intervention might be effective but randomization into the control arm will not cause any lower quality of care as patients in the control arm will get standard stroke care plus standardized information material.

### Outcome measures

#### Primary outcomes

- Change in Patient Health Questionnaire-9 (PHQ-9) score from Baseline (V1) to day 84 (V2).

#### Secondary outcomes

- Change in Patient Health Questionnaire-9 (PHQ-9) score from Baseline (V1) to days 84, 180 (V2, V3) and 365 (V4).
- Change in EuroQol- 5 Dimension (EQ-5D) score from Screening (V0) to days 84, 180 and 365 days (V2-V4).
- Change in modified Rankin Scale (mRS) from Screening (V0) to days 84, 180 and 365 (V2-V5).
- Return to work from Screening (V0) to days 84, 180 and 365 days (V2-V4).
- Incidence of major cardiovascular events defined as nonfatal stroke (ischemic or hemorrhagic), nonfatal myocardial infarction (including acute coronary syndrome requiring emergency vascularisation), and vascular death (i.e. sudden cardiac death and death from acute myocardial infarction, ischemic or hemorrhagic stroke, heart failure, cardiovascular procedures, pulmonary embolism, or peripheral artery disease) within one year of the index event.
- Recurrent ischemic or hemorrhagic stroke or TIA (transient ischemic attack; defined as transient neurological deficit < 24 h and absence of DWI (Diffusion-weighted imaging) positive lesions on MRI (Magnetic resonance imaging)) within one year of the index-event.
- All-cause mortality at 14 months.

Regular mortality follow-ups will be done for gathering information on deaths in the study sample. Death-certificates will be provided by local health authorities, causes of death will be specified using the International Classification of Diseases (ICD)-11.

### Eligibility criteria

All patients with acute ischemic or hemorrhagic stroke admitted to the Neurology Department at the University Hospital of Augsburg will be screened for depressive symptoms during the first days of their hospital stay as part of the DESIE (Depression bei somatischen Erkrankungen) Study. Here, a PHQ-9 cut-off score of ≥ 10 was found to have a high sensitivity (91%) and specificity (89%) for major depression and any other depression diagnosis (78% / 96%) [18]. Patients are eligible for inclusion irrespective of whether the index event was a first or recurrent event. Ischemic stroke is ascertained using criteria based on clinical and/or imaging features. Patients with transient ischemic attacks are excluded from the study, as they do not have persisting stroke symptoms. These patients might probably not benefit from the intervention and might distort effects between the intervention or control group. For study inclusion, patients must give their written informed consent. Patients, who cannot give informed consent, e.g. because of dementia or aphasia, are not eligible. Neither are patients with legal guardians, those with premorbid mRS of > 2, or patients with a shorter life-expectancy than the expected duration of the trial, e.g. due to malignancy or other severe diseases.

#### Inclusion criteria

- Patients with acute ischemic or hemorrhagic stroke.
- Acute neurological treatment in the University Hospital Augsburg.
- Age ≥ 18 years.
- Signed informed consent.
- PHQ-9 ≥ 10 at screening.

#### Exclusion criteria

- Patients with transient ischemic attack.
- Malignancy or other severe disease with life-expectancy less than the expected duration of the clinical study.
- Inability of giving informed consent (e.g. aphasia, dementia).
- Patients with legal representatives.
- Premorbid modified Rankin Scale > 2.

Recruitment for DISCOVER already began in March 2024 and is planned until February 2025.

## Contacts

Investigator initiated trial; M. Ertl (Principal Investigator); Michael.Ertl@uk-augsburg.de.

## Perspective

The purpose of this interventional study is to evaluate the efficacy of the telepsychological application of Behavioural Activation (BA) -therapy for stroke patients with post-stroke depression (PSD), after screening them for depressive symptoms.

To screen all eligible patients for PSD limits the known risk of missing this significant complication [3]. Here the Patient Health Questionnaire (PHQ)-9 can be used to screen for PSD, regardless of age, gender or ethnicity [18]. If proven to be helpful, an implementation into disease management programs for stroke patients, which are gaining increasing interest and are endorsed by the German Stroke Society, could be done.

Besides the benefits of accessibility and scalability of telepsychological treatment, is growing evidence, that it is not only feasible, but may also have similar or in some instances even better effects compared to classical face-to-face therapy [19]. However, to our best knowledge, the application of telepsychological interventions for PSD has not been investigated before. In this context, BA represents an innovative and evidence-based advancement of classical behaviour activation and can easily be applied in a remote manner.

Besides the new way of applying the treatment, the 12-week treatment period is similar to the common durations [10, 14], thus improving comparability to the existing literature.

The offered standardized information material about PSD helps patients to understand the disease and its significance, thus increasing their involvement.

Evaluating the secondary outcomes will help us comprehend the context of potential treatment effectiveness. Here, the health-related quality of life (HRQoL) is particularly relevant. Assessing the HRQoL can be done by questionnaires. Scores, such as the EuroQol five-dimensions (EQ-5D) have been tested and validated for HRQoL after stroke [20].

This may be a new step towards digital medical treatment options for people who need more tailored health care after stroke, but face shortages of qualified staff and poor accessibility in some areas. This could enhance the quality of life of stroke survivors, their rehabilitation success, and general socioeconomic aspects of chronic stages of stroke.

## Data Availability

The datasets used and/or analysed during the current study are available from the corresponding author on reasonable request.

## Abbreviations

ADL: activities of daily living
AE: adverse events
BA: Behavioural Activation
BP: blood pressure
DALYs: Disability-adjusted life-years
DESIE: Depression bei somatischen Erkrankungen
DFG: Deutsche Förderungsgemeinschaft
digiBRAVE: digitale Bayerische (Früh-)-Diagnostik-, Prävention und Therapieprogramm Depression
DWI: Diffusion-weighted imaging
EQ-5D: EuroQol- 5 Dimensions
HR: heart rate
HRQoL: health-related quality of life
ICD: International Classification of Diseases
ITT: intention-to-treat
M: mean
MFB: mindfulness based intervention
mITT: modified intention-to-treat
MRI: Magnetic resonance imaging
mRS: modified Rankin scale
NIHSS: National Institutes of Health Stroke Scale
PHQ-9: Patient Health Questionnaire-9
PP: per protocol
PSD: Post-stroke depression
SAE: severe adverse events
SD: standard deviation
TIA: transient ischemic attack
